# Dendritic cells in cancer immunology

**DOI:** 10.1038/s41423-021-00741-5

**Published:** 2021-09-03

**Authors:** Theresa L. Murphy, Kenneth M. Murphy

**Affiliations:** Department of Pathology and Immunology, Washington University in St. Louis, School of Medicine, St. Louis, MO 63110 USA

**Keywords:** Dendritic cells, transcription factors, cross-presentation, tumor rejection, CD4 help, Cellular immunity, Conventional dendritic cells

## Abstract

The clinical success of immune checkpoint therapy (ICT) has produced explosive growth in tumor immunology research because ICT was discovered through basic studies of immune regulation. Much of the current translational efforts are aimed at enhancing ICT by identifying therapeutic targets that synergize with CTLA4 or PD1/PD-L1 blockade and are solidly developed on the basis of currently accepted principles. Expanding these principles through continuous basic research may help broaden translational efforts. With this mindset, we focused this review on three threads of basic research directly relating to mechanisms underlying ICT. Specifically, this review covers three aspects of dendritic cell (DC) biology connected with antitumor immune responses but are not specifically oriented toward therapeutic use. First, we review recent advances in the development of the cDC1 subset of DCs, identifying important features distinguishing these cells from other types of DCs. Second, we review the antigen-processing pathway called cross-presentation, which was discovered in the mid-1970s and remains an enigma. This pathway serves an essential in vivo function unique to cDC1s and may be both a physiologic bottleneck and therapeutic target. Finally, we review the longstanding field of helper cells and the related area of DC licensing, in which CD4 T cells influence the strength or quality of CD8 T cell responses. Each topic is connected with ICT in some manner but is also a fundamental aspect of cell-mediated immunity directed toward intracellular pathogens.

## Introduction

We approach this review on cancer immunology from the viewpoint of conventional dendritic cells (cDCs). We present three threads of DC biology that relate directly to how DCs support effective T cell responses to tumorigenesis. Each part relates to a subset of cDCs historically called by various terms, such as CD8α^+^ DCs, CD103^+^ cDCs, or Xcr1^+^ cDCs [1–3]. Currently, the term cDC1 is used to refer to all of these types of cDCs [4] because each of the previously used terms referred to the same lineage of conventional DCs controlled by a single genetic pathway and can be eliminated by specific disruptions to that pathway [5–7]. The rationale for this focus is the critical function cDC1s play in antitumor immunity. The areas covered here are experimental, not clinical, and focus on recent work, primarily studies with mouse models and experimental tumor systems. The purpose of this review is to highlight recent basic advances in the area of cDC1 development and function. First, we cover work that led to a specific definition of the transcriptional program defining cDC1s, which distinguishes cDC1s from other types of DCs. Although aspects of this work began in 2008 [5], the finding that cDC1s were “Batf3-dependent” was not satisfactorily explained until relatively recently, after extensive mechanistic work was performed, largely with T cells and B cells [8–10]. Second, we cover work in the area of cDC1 antigen processing of tumor antigens that can be recognized by CD8 T cells, called cross-presentation. This topic has a long history, not without controversy, and remains an area of active and ongoing research. Finally, we cover work dealing with the mechanisms by which CD4 T cells “help” to prime CD8 T cells, another topic with a surprisingly long history and important implications for the design of cancer vaccines, since antigens presented by MHC class II molecules can impact the effectiveness of antigens presented by MHC class I molecules.

## General background in DC biology

Initially discovered in the 1970s by Steinman as being cells with a powerful ability to stimulate allogeneic T cell responses [11], DCs were later recognized as integral to activating antigen-specific responses. By the 1990s, it was recognized that “DCs” comprise a variety of subtypes that were initially distinguished by distinct surface markers. A general classification was used to distinguish classical (or conventional) DCs (cDCs) from plasmacytoid DCs, which are currently thought to mediate responses by their cytokine secretion and not to participate directly in the presentation of antigens to T cells [12]. More recently, the underlying transcriptional basis for the distinctions among DC subsets was discovered [13], and proteins that function as lineage-determining factors or as terminal selectors in these lineages were identified [14]. These discoveries increased the appreciation that DCs constitute a group of closely related cell types, and this longstanding research, extending back to the 1990s, remains an active area of inquiry.

**Fig. 1:**
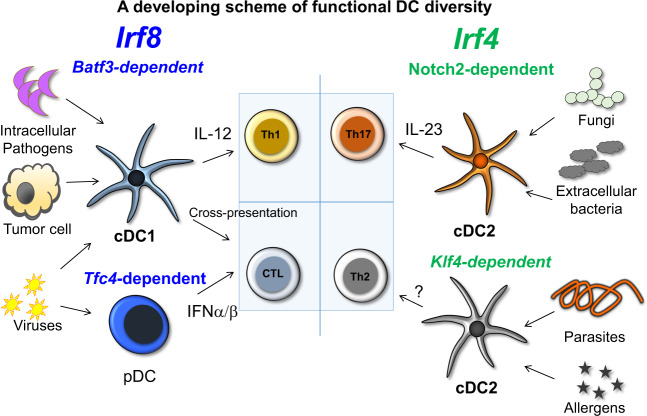
Proposed scheme showing functional DC diversity. The genetic basis of functional DC diversity is shown with IRF8-dependent types, cDC1s and pDCs, shown on the left. Both cDC1s and pDCs function in antiviral immunity, with pDCs contributing type I interferon and cDC1 priming CD8 T cells through cross-presentation of antigens. cDC1s are also active in some intracellular infections, such as in *Toxoplasma gondii*, where their IL-12 production in response to pathogens is key for host defense. cDC1s also prime CD8 T cells to recognize tumor cell antigens through cross-presentation. cDC2s can aid in protection from other pathogens, and evidence suggests that defense against some pathogens, such as *Citrobacter rodentium*, relies on a Notch2-dependent program, while defense against helminths relies on KLF4 action in cDC2s.

The molecular and transcriptional analysis of DC subsets allowed the development of several experimental systems that have enabled selective in vivo ablation of some subsets, and these systems have helped identify specialized DC functions. Although many studies in the area of tumor immunology are not based on these experimental systems, certain studies have focused on the function of these DC subsets in various infectious disease models (Fig. 1) [7]. It is clear that cDC1s cross-present tumor-associated antigens in a manner similar to their presentation of viral antigens, leading to CTL responses. Overall, these studies have led to the proposal that pDCs and various subtypes of cDCs specialize in promoting alternative ‘modules’ of immunity that specifically match a pathologic challenge [15]. These studies have informed working models in which various DC subsets promote responses that mount the best defense against specific pathogen (or tumor) types (Fig. 1). The mechanisms underlying the promotion of different types of responses are not firmly established in all cases, and therefore, the investigation into these mechanisms remains an active area of research. DCs can act both on innate lymphoid-like cells (ILCs) and T cells and can promote either a cell-mediated response by stimulating ILC1s/Th1 cells/CTLs that respond to extracellular signals, or by stimulating ILC3/Th17 cell-type responses, or finally by stimulating a response to helminths through unknown pathways. While this scheme is continuously being modified as new experimental systems emerge, the essential role of the cDC1 subset in driving cell-mediated responses based on CD8 T cell priming appears to be solidly established. Coordination between DC subsets and the immune module they stimulate suggests that genetic programs regulating DC diversification preceded the emergence of RAG-dependent adaptive immunity. Our purpose in this review is to describe the genetic program underlying the cDC1 subset.

Immune checkpoint therapy (ICT) used in cancer is based on monoclonal antibodies targeted to the checkpoint molecules CTLA-4, PD-1, and/or PD-L1 to augment antitumor immune responses, even in cases of low tumor immunogenicity, and requires cDC1s action to be effective [16]. DCs are drivers of innate and adaptive immune responses, not only in cancer but also in infections. Recent studies have demonstrated the significance of F (B7-H1) expression and function in human and mouse APCs, including DCs [17–19]. Much of the current knowledge on DC function was obtained from studies with mice based on surface marker expression, transcriptomics, and analyses of DC development and function [4, 13]. Human DCs express different surface markers than mouse DCs [13, 20, 21] but are thought to rely on the same developmental transcriptional programs and partake in the same functional activities as their mouse counterparts [22].

cDCs are potent antigen-presenting cells (APCs) during immune responses and comprise two major lineages: the cDC1 and cDC2 subsets. The cDC2 subset is important for the induction of Th17 and Th2 cell immune responses and thus regulates immune responses to extracellular pathogens, parasites, and allergens [23–29]. The cDC1 subset is important for type 1 immunity in response to intracellular pathogens through their production of interleukin 12 (IL-12) [6, 30, 31]. In addition, human cDC1s recruit neutrophils to help clear bacterial and fungal infections in skin [32, 33]. pDCs are developmentally related to cDCs, but the former does not play a major role in antigen presentation [34, 35].pDCs produce type I interferon during viral infections and are in certain respects related to innate lymphoid cells [36].

### Dendritic cell subsets and their development

**Fig. 2:**
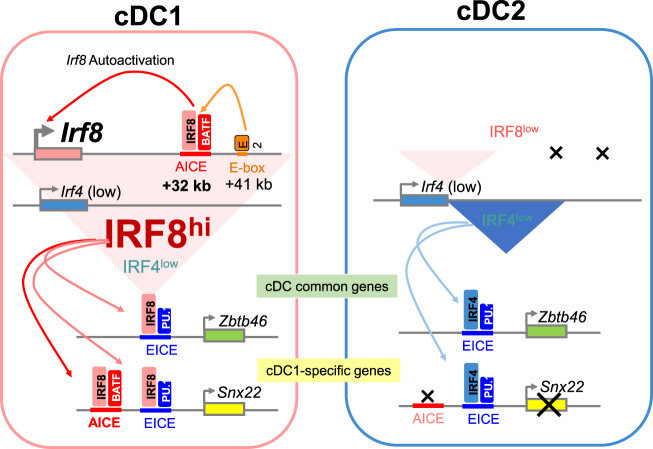
The basis for cDC1/cDC2 diversity lies in the activation of an AICE-dependent gene program. In cDC1s, the Irf8 gene undergoes autoactivation through the action of an IRF8:BATF3 complex binding to an enhancer located +32 kb downstream of the Irf8 promoter. This autoactivation maintains high levels of IRF8 expression in the maturing cDC1 of a specific progenitor. In contrast, in cDC2s, the level of Irf8 is not maintained, and both IRF4 and IRF8 are expressed together but at much lower levels. This level of IRF4 and IRF8 expressed in cDC2s is sufficient to activate the EICE-dependent program that controls the expression of genes expressed in cDCs, both in cDC1s and cDC2s, such as *Zbtb46*. However, in cDC1s, the expression of IRF8 is much higher and sufficient to activate the program of genes that also require occupation of AICE cis-acting elements, such as the cDC1-specific gene *Snx22*. In *Irf4* deficiency, a low level of IRF8 protein is sufficient to maintain the expression of some common cDC genes, but the induction of certain genes is inadequate for induction of some certain responses.

In this section, we answer two puzzling questions. First, how are cDC1 and cDC2 different? IRF4 and IRF8, the factors typically used to distinguish cDC1 and cDC2, are very similar, and little evidence indicates that they bind distinct DNA sequences or interact with different partners. Second, why is *Batf3* required for cDC1 development, but not for cDC2 development? *Batf3* is highly DC-specific and is expressed by both cDC1 and cDC2s but no other cells. Confusion over the cDC1 specificity of *Batf3 *manifests through suggestions by some to use *Batf3* as a Cre driver for cDC1-specific deletion or references to cDC1s as ‘Batf3^+^ cDCs’. In fact, *Batf3* would likely drive Cre expression in both cDC1s and cDC2s. More accurately, cDC1s are sometimes referred to as *Batf3*-dependent DCs, but until recently, an explanation for this dependence was lacking. The following discussion leads to the formulation of the model shown in Fig. 2.

## Conventional DCs and monocyte-derived DCs are distinct types of cells

We first need to clarify some unique aspects of in vitro model systems, particularly to draw a distinction between conventional DCs and monocyte-derived DCs. cDCs can be generated in vitro from bone marrow progenitors cultured in the presence of Flt3L [37]. In culture, Flt3L bone marrow secretes cells that appear to be authentic lineages of cDC1s, cDC2s, and pDCs. The literature includes articles in which the term DCs is used to refer to cells derived in vitro from monocytes (MoDCs) or bone marrow progenitors (BMDCs) cultured with GM-CSF with or without IL-4 [38–40]. While MoDCs and BMDCs are used extensively and have been used as primary in vitro models for a long time, recent work shows that they produce heterogeneous populations comprising macrophage-like and DC-like cells [41]. The DC-like cells produced in these models do not fit strictly into the definition of either the cDC1s or cDC2s obtained in vivo or in Flt3L-enriched cultures. Lineage tracing suggests that in vivo, the cells derived from monocytes under inflammatory conditions do not express the cDC marker *Zbtb46* [42], in contrast to their in vitro counterparts, and therefore may not reflect the extent of differentiation that can be induced in vitro. The function of DCs derived from monocytes in tumor immunity and ICT is currently unclear. While extensive effort has been directed toward adapting them for therapeutic use, we do not cover these cells in this review. Here, we focus on cDCs and Flt3L-derived DCs

## Studies of DC progenitors reveal insights into the molecular basis of cDC divergence

It was initially thought that cDC1s and cDC2s were derived from a CDP, also called a ‘pre-cDC’, and defined as Lin− CD135^+^MHCII^−^CD11c^+^ DC [43]. Later, a distinct progenitor for each cDC lineage was identified and found to arise from a CDP [44–47] One method of identifying pre-cDC1 and pre-cDC2 progenitors in bone marrow relied on reporter mice expressing GFP under control of the endogenous *Zbtb46* gene [48]. Later, it was found that CD226 can be used in place of the Zbtb46-GFP reporter to identify pre-cDC1s [49]. The original identification of pre-cDC1s included heterogeneous CD115 (MCSF-R) expression [45], but later, pre-cDC1s were found to originate within the CD115 + DC fraction [49], arising from a CDP (a Kit^int^ population), and CD115 expression was found to be reduced before the cDC1s moved from the BM into the circulation [45]. Pre-cDC2s were also found in the CD115^+^ fraction of pre-DCs, while the CD115^−^ a fraction of pre-cDCs was found to develop into both pDCs and cDC2s.

An important discovery arising from this work on progenitors revealed the identity of a pre-cDC1 progenitor which surprisingly showed normal development in *Batf3*-deficient mice. The pre-cDC1 and pre-cDC2 progenitors were separate populations which generate cDC1 or cDC2s respectively when cultured in vitro with Flt3L [45]. In *Batf3*^−/−^ mice, the pre-cDC1 progenitor was clearly distinguishable from the pre-cDC2 progenitor, but when cultured in Flt3L, the pre-cDC1 progenitor gradually started to express the surface markers of a cDC2, and importantly, the level of IRF8 expression in the progenitor was reduced to a low level that is typical of the cDC2 lineage. This change in IRF8 expression is a strong indication that the level of IRF8 might be particularly significant and that *Batf3* might play a role in sustaining IRF8 expression in mature cDC1s but not in progenitors.

## BATF3 stabilizes *Irf8* autoactivation at the +32 kb *Irf8* enhancer during cDC1 commitment

*Irf8* was the first factor found to be required for cDC1 development [50–53]. *Irf8*^*–/–*^ mice not only lack cDC1 in the periphery but also show a significant reduction in the number of CDPs, and they exhibit other defects, such as an increase in the number of neutrophils [53–55]. Work on the mechanism of action of BATF family members, such as *Batf*, *Batf2,* and *Batf3*, led to the discovery that, in a unique feature of these sub-family, AP-1 factors interact with their common leucine zipper (LZ) domain to form two unique interactions [8]. First, similar to other AP-1 factors such as Fos, BATFs form a heterodimer with Jun, and interactions of this dimer to AP-1 motifs have been clearly established. A second unique interaction was discovered by several studies examining the global binding of BATF in T cells: [8, 56–58] an extensive overlap among binding sites for BATF and for the factor IRF4, which is also expressed in T cells. In-depth studies revealed that the molecular basis for BATF interactions rely on residues in the LZ domain that face away from the surface where Fos interactions are mediated [8], thus forming a bridge linking the IRF factor to the AP-1 factor. At the level of DNA binding, a novel motif, called the AP-1/IRF consensus element, AICE, was found to mediate the unique transcriptional program of BATF factors in various cells.

With this new mechanistic insight, the role of *Batf3* in cDC1 commitment and stabilization of IRF8 expression were examined. Through a series of studies using ChIP-seq and ATAC-seq, the *Irf8* gene locus was found to contain several potential enhancer elements [45, 59]. One enhancer +41 kb relative to the *Irf8* transcriptional start site (TSS) was found to be highly active in pDCs and to bind E proteins. This enhancer also seems to be active in CDPs and is required for the increase in the level of IRF8 expression that occurs as CDPs emerge from MDPs, although whether E proteins mediate this transition is unclear. A specific cDC1 progenitor develops from a CPD even in the absence of *Batf3*, as described above. At this stage, the *Irf8* enhancer at +41-kb enhancer is abandoned and an enhancer located +32 kb from the TSS is occupied. In this region, there is a cluster of several AICEs that can bind to the BATF3:IRF8 complex, as indicated by IRF8 and BATF3 Chip-seq [45, 59]. Here, BATF3 is required for cDC1 commitment, as the BATF3 complex that includes IFR8 is necessary for further cDC1 development. This requirement was evident upon deletion of a 150-bp region containing the majority of these AICEs, which led to the expression of the same phenotype as that acquired upon *Batf3* deficiency: cDC1-specific identity is established but is subsequently lost. In addition, IRF8 levels gradually decrease to the low levels found in cDC2 cells.

## Additional targets of BATF3 in cDC1s are required for tumor rejection

*Batf3* plays other roles in cDC1s in addition to supporting IRF8 expression. In a major contribution to cDC1 development, BATF3 cooperates with IRF8 to support high IRF8 expression via the +32 kb *Irf8* enhancer. Since cDC1s are essentially absent in *Batf3*^*–/–*^ mice [5, 6], it was not easy to determine whether BATF3 also acts in the transcription of other genes selectively expressed in cDC1s, such as XCR1 or CLEC9A. cDC1s can reappear under inflammatory conditions in *Batf3*^*–/–*^ mice [8], but this system is also not useful for the analysis of BATF target genes. Even compensation through *Batf* and *Batf2* expression [30], which restores the cDC1 level in the absence of Batf3, was not sufficient for identifying BATF3 target genes. Thus, the identity of other genes that might depend on the expression of *Batf3* to imprint cDC1 identity, which is required to promote the various functions required for antitumor CD8 responses, remained largely unknown.

However, recent progress has been made in identifying *Batf3* targets in cDC1s [60]. Conveniently, IRF8 appears to be artificially maintained even in the absence of BATF3 in when 3 copies of an *Irf8* transgene are expressed [61]; this transgene was initially generated for use as a fluorescent reporter for *Irf8*, expressing the VENUS reporter under the control of a large segment of the genomic Irf8 locus. In the reporter line, 3 *Irf8* copies were incorporated, and whether intentional or inadvertent, the expression of these copies is sufficient to maintain IRF8 production at a level that maintained the specified pre-cDC1 progenitor in a state of high IRF8 expression during its lifetime in vivo. Remarkably, this state was maintained even when crossed onto a *Batf3*-deficient background [60]. This observation, made somewhat by serendipity, nonetheless allowed for the identification of cDC1-specific genes that require BATF3 for their expression, similar to *Irf8*. The number of these BATF3-dependent genes was surprisingly small, with 10 that strictly require both IRF8 and BATF3 to mediate their expression. While the *Batf3*^*–/–*^ cDC1s that arose in this model were able to cross-present cell-associated antigens effectively, the mice harboring these cDC1s showed unattenuated tumor development. This outcome indicates that some combination of BATF3-target genes likely plays an important role in the cDC1 biology required for fully functional in vivo behavior. Currently, these genes and their functions are unknown.

## cDC1 identity relies on the transcriptional engagement of an AICE-dependent gene program

Early studies proposed that the cDC1 and cDC2 dichotomy was based on differential activity of the transcription factors IRF8 and IRF4. Several studies showed a role for these factors in DCs. Mice lacking IRF8 also lacked CD8α^+^ cDCs, and mice lacking IRF4 also lacked CD4^+^ cDCs [62]. These outcomes were initially interpreted as IRF8 and IRF4 being strictly required for the development of these lineages. One study revealed that CD8α^+^ cDCs were identical to CD103^+^ cDCs, which were found to be expressed in tissues found to be lacking CD8α^+^ cDCs, but later studies showed that cDC1s constitute a lineage in which expression of certain markers can vary based on their location and maturity [6, 31]. It is now clear that all of the various subsets of cDC1 in all locations are absent in *Irf8*-deficient mice, but in the case of cDC2s, the situation is more nuanced [63]. Kovats and colleagues discovered that *Irf4* deficiency does not lead to the elimination of the cDC2 lineage per se, but in these mice, splenic cDC2s lacked CD4, a marker of cDC2s in the spleen [63]. However, other cDC2 markers were expressed, showing that the lineage was present, and cDC2 responses were found to be altered in the absence of IRF4.

Later, the discovery of substantial overlap in the target specificity of IRF4 and IRF8 offered an explanation for these findings [64]. Mice lacking both *Irf4* and *Irf8* have no cDCs in the spleen or peripheral tissue. IRF4 and IRF8 are highly homologous and have both redundant and unique roles in DC development and function. *Irf4* is expressed at a higher level in cDC2s than in cDC1s. *Irf4*-deficient mice maintain all cDC and pDC lineages in the spleen and retain some but not all cDC2 subsets in the lung and small intestine [23, 65]. *Irf4* regulates some cDC2 functions, such as migration, through the control of CCR7 expression [63, 66, 67].

IRF8 plays a major role in myeloid cells, including monocytes and DCs, but plays a less prominent role in lymphocyte function. IRF8 is very highly expressed in cDC1 and pDCs and is expressed at a low level in cDC2s. *Irf8*^−/−^ mice completely lack pre-cDC1s as well as cDC1s in spleen and peripheral tissues [62, 64, 68]. These mice have fewer CDPs that give rise to cDC2s, which likely develop through a compensation mechanism mediated by *Irf4*. *Irf8* is not required for pDC development but is required for the expression and functions of some pDC markers [14]. *Irf8* expression can be detected using *Irf8*-GFP reporter mice during hematopoiesis as lympho-myeloid primed progenitor (LMPP), a multipotent progenitor upstream of CMPs [69]. LMPPs and other progenitors that express *Irf8* predominantly become cDC1s and display Irf8-dependent epigenetic and transcriptional programs.

Both IRF4 and IRF8 interact with several other TFs in DCs and progenitors, including PU.1, BATF factors, and Cebpα. PU.1 recruits both IRF4 and IRF8 to Ets-IRF composite elements (EICEs) [70–72], which are cis-elements important for the determination of DC identity, including in the control of *Zbtb46* expression [64]. IRF4 and IRF8 bind in combination with the AP1 family TFs BATF1, BATF2, and BATF3 to AP1-IRF composite elements (AICEs) [8, 56–58], which are specific and important for cDC1 development. *Irf8*-deficient mice have an increased number of neutrophils because they lack the inhibitory function of IRF8 on Cebpα-DNA-binding activity [73]. No reports to date have indicated that IRF4 can also bind to Cebpα. Some of the apparent specificity of IRF4 and IRF8 in cDCs is due to the level of expression, not the protein sequence. For example, both IRF4 and IRF8 can restore cDC1 and cDC2 development in cultures of doubly deficient *Irf4*−/− *Irf8*−/− BM progenitors, and both are capable of supporting cDC1-mediated cross-presentation when their expression levels are sufficiently high [64].

### The mechanism of cDC1 cross-presentation in antitumor responses

This section answers questions regarding the cells and mechanisms of cross-presentation in vivo that lead to tumor rejection by CD8 T cells. The determination of the cells that perform this function is very importance because extensive efforts have been devoted to cell-based immunotherapy that activates antitumor immunity and that relies on in vitro production of MoDCs, with limited success [74, 75]. In addition to the identities of the specific cells involved, understanding the molecular pathway of cross-presentation may lead to enhanced cell-based immunotherapies. Remarkably, what we now call cross-presentation was discovered long before the mechanism T-cell antigen recognition was understood [76]. Bevan’s original term, ‘cross-priming’, referred to the priming of CD8 T cells to donor minor allo-antigens, which was restricted by the host’s specific MHC alleles. In this definition, ‘cross’ indicated crossing between MHC alleles and exogenous or endocytic antigens cross-presented for intracellular processing. Despite the limits to measuring polyclonal responses to minor antigens that had not yet been identified, Bevan’s interpretation was correct in that exogenous (donor-derived) antigens are processed by host APCs for recognition by host CD8 T cells. Several cell types, including cDC1, Mo-DCs and GM-DCs, can cross-present antigens, particularly in vitro. However, which cell types can and do carry out the function of cross-presentation in vivo remain to be determine.

## Germline *Batf3* deficiency eliminates antitumor immunity

*Batf3*^−/−^ mice were the first in vivo models of cDC1 ablation, and they were useful in confirming the in vivo role of cDC1s in providing cross-presentation of various antigens of pathogens and tumor to CD8 T cells [5, 77–90]. In the original study, mice with *Batf3* germline deficiency were examined [5] to ensure that different interpretations were possible. On the one hand, cDC1s might be required for tumor rejection because they are the only APCs that carry out cross-presentation in vivo to a significant extent, as originally claimed. On the other hand, the absence of cDC1s may be a red herring, merely a correlated finding, and the lack of immune response may be caused by *Batf3* deficiency in cells residing in *Batf3*^−/−^ mice, such as MoDCs. These issues are not simply academic but are actively debated.

These alternative possibilities were not identified until relatively recently. Some clues in early studies indicating that cDC1s might be required for tumor rejection were based on analysis of the molecular requirement of cross-presentation in different cells. First, it was found that neither the development of GM-DCs and Mo-DCs nor their in vitro cross-presentation activity depended on *Batf3* [91]. This finding indirectly suggested that these cells would not be affected in *Batf3*^−/−^ mice. Hence, if these cells participate in priming CD8 T cells in vivo, then they presumably can prime CD8 T cells in *Batf3*^−/−^ mice. Further, germline and conditional deletion of the cDC1-specific *Rab43* gene reduced cross-presentation by cDC1s but not by MoDCs, suggesting that different cellular pathways are activated for this process in these two types of cells.

## Precise genetic models pinpoint cDC1s as sites of in vivo cross-presentation in tumor rejection

Evidence supporting the roles of cDC1s but not MoDCs in cross-presentation in vivo is based on two genetic models: the cDC1-specific *Xcr1*-Cre deletion strain [92] and germline WDFY4 deficient strain [93]. Cross-presentation has been studied primarily using MoDCs and GM-DCs with various forms of antigens [94]. These studies identified two major models of cross-presentation that involve either transport of exogenous antigen to the cytosolic proteasome before peptide loading in the endoplasmic reticulum (ER) [95–99] or peptide loading directly on phagosomes upon fusion of phagosomes with vesicles containing the peptide-loading complex, potentially depending on the SNARE family member Sec22b [100–103]. In particular, cross-presentation by MoDCs has been attributed to the action of NOX2, Rac2, Rab27a, IRAP, Rab3b/c, mannose receptor, TFEB, Sec61 and Sec22b [102–112]. Although multiple studies have identified a number of genes that are proposed to mediate cross-presentation in MoDCs, to date, the majority of these candidates have not been tested in vivo for their role in cross-presentation or antitumor immunity. The exception is *Sec22b*, which was found in one study to contribute to the efficiency of immune responses only in the setting of ICT but was not required in another study.

However, two in vivo models have recently indicated that MoDCs are unlikely to cross-present antigens in vivo and that this function is carried out by cDC1s. First, a model of cDC1-specific gene deletion was generated by placing Cre under the control of the endogenous *Xcr1* locus [92], which is innately highly cDC1-specific [3]. Crossing *Xcr1*-Cre mice to mice with a conditional allele of MHC class I led to the generation of mice in which MCH I is expressed on all cells except cDC1s [92]. These mice were incapable of rejecting tumors or priming antigen-specific CD8 T cells to recognize cell-associated antigens. This result seems to exclude an important role for MoDCs in priming CD8 T cell responses in these models, since these cells still express MHC class I, but in vivo responses to a tumor or cell-associated antigens were eliminated.

Further evidence was shown by studies into the mechanism of cross-presentation itself [93]. Based on evidence that different genetic and molecular pathways are activated in MoDCs and cDC1s during cross-presentation [91, 113], a CRISPR/Cas9 genetic screen was used to identify genes required for this activity specific to primary cDC1 cells [93]. The gene *Wdfy4* was found to be required for cross-presentation of cell-associated antigen by cDC1s. *Wdfy4* is expressed in both cDC1s and cDC2s. *Wdfy4*^−/−^ cDC1s failed to cross-present cell-associated antigens in vitro, and importantly, *Wdfy4*^−/−^ mice failed to reject immunogenic sarcomas. Although GM-DCs also express *Wdfy4*, it was not required for cross-presentation.

**Fig. 3:**
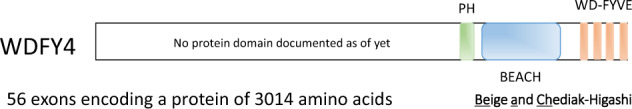
WDFY4 is a BEACH domain-containing protein required for cross-presentation by cDC1s in vivo and in vitro. The WDFY4 protein shares similarity with other members of the BEACH domain-containing protein family. The PH (green) and BEACH (blue) domains comprise nearly one-third of a protein, lying in the carboxy-terminal region along with other domains, namely, WD40 and FYVE repeats (brown). The first approximately 2000 amino acids lack defined domains, although ongoing research suggests that this region contains at least one larger armadillo domain. The precise mechanism by which this protein plays a required role in cross-presentation is unknown despite high profile speculations.

WDFY4 is a BEACH-domain-containing protein (BDCP), of which there are nine mammalian family members, which typically also contain a PH-like domain and WD repeats (Fig. 3) [114–116]. Several BDCPs are associated with human diseases [114]. For example, mutations in the BDCP *Lyst* cause Chédiak-Higashi syndrome, which is a primary immunodeficiency-related disease characterized by defective neutrophil phagolysosome formation and cytotoxic T cell degranulation [117, 118]. Mutations in *Lrba*, another BDCP, result in altered trafficking of CTLA4 between endosomes and lysosomes mediated by the clathrin adaptor AP-1. This alteration causes increased trafficking to lysosomes, leading to a reduction in CTLA4 levels that are insufficient for the normal control of regulatory T cells [119, 120]. In this case, the therapeutic immune function could be restored by inhibiting lysosome activity using the drug chloroquine.

WDFY4 is conserved across species, and WDFY4 mutations in humans were found to be associated with various immunological disorders in a GWAS [93, 121–123]. A BDCP can act as a scaffold for intracellular vesicular fission and fusion. For example, WDFY3, the closest WDFY4 homolog, regulates the recruitment of polyubiquitinated protein aggregates to autophagosomes by interacting with p62, Atg5, Atg12, Atg16L, LC3, and TRAF6 [124–127].

Cross-presentation of cell-associated antigens does not involve autophagy [128] but does involve vesicular trafficking. WDFY4 is localized to submembrane endosomes, and initial studies suggested that it may interact with endocytic and cytoskeletal machinery and likely plays a role in vesicular trafficking during cross-presentation, but the precise interacting proteins are currently unknown.

The challenge to further analysis of the mechanism involving WDFY4 in cross-presentation relates to the size of the protein (Fig. 3). WDFY4 comprises more than 3000 amino acids, its gene comprises more than 50 exons spread across 100 kb of genomic length, and its cDNA, at approximately 10 kb, is at the limit of that easily expressed by retroviruses. Nonetheless, it will be important to identify the interacting partners of WDFY4 to uncover the pathway activated in cDC1 during the processing of tumor-derived antigens, as this information may yield insights into how to obtain cross-presenting cells that may be useful in a cell-based immunotherapy regimen. Additionally, 9 BDCPs are typically expressed in different types of cells. It remains to be determined whether they compensate for each other, and if they do, the extent of their compensation. In particular, it is unknown whether WDFY3 has the same function as WDFY4 when expressed in cDCs, and similarly, do subsets of Lyst and LRBA compensate for each other? Information regarding the molecular basis of all these proteins is sparse, and more information about their interchangeability may help resolve issues about the underlying mechanisms of their actions.

### cDC1s offer autonomous platforms for priming CD8 T cells as facilitated by CD4 T cells

In this section of the review, we summarize recent work related to the question of how CD4 T cells ‘help’ CD8 T cells respond to tumors. This area of inquiry can be broken down into specific parts: 1) which type of APC primes CD4 helper cells? 2) What is the cellular source of CD4 helper cells? and 3) By what mechanism does CD4 helper cells influence CD8 T cells? Similar to the cross-presentation field, the ‘helper’ T cell field has a long history and has evolved greatly in its sophistication over time. A major motivation to understand helper T cells more deeply arises from recent findings in experimental studies showing mechanisms of ICT. For example, one recent study showed that tumor rejection is enhanced by the combination of antigenic epitopes presented by both MHC class I and MHC class II [129], a finding with direct implications for tumor vaccine design. Earlier, the same group of researchers found that in systems in which tumor rejection required ICT, rejection relied on the cDC1 axis of immunity [130].

## What APCs prime CD4 T cells to help in antitumor CD8 responses?

Previous studies have suggested that CD4 T cells are exclusively activated by cDC2s. In some studies, cDC2s were found to have a superior capacity for MHC-II antigen processing and presentation relative to cDC1s when soluble ovalbumin (OVA) or OVA coupled to antibodies targeted to Fc or surface receptors was used as the antigen [131, 132]. A particular strength of these studies is the direct comparison of CD4 and CD8 T cell proliferation in vivo, but a caveat is the particular form of antigen delivery used in these studies may not be applicable in all cases. This division of labor between DCs and T cells is not absolute, since the same group reported that cDC1 can present antigens on MHC class II molecules [133]. In summary, these studies led to the general model that CD4 T cells are required for optimal CD8 T cell responses to cell-associated antigens but not to soluble antigens. Further, CD4 T cells help may be facilitated by CD40, although the target for this interaction has not been clearly identified, as most studies reporting this finding relied on antibody blockade. Finally, whether the CD4 interaction with its target cell is antigen-specific was not firmly established. However, later studies seem to agree with this interpretation, although the findings are based on indirect results. For example, in *Irf4*^–/–^ mice, which exhibit impaired cDC2 migration [63], CD4 T cell responses to allergens and fungal infections in the intestine and lung were attenuated [23, 134], consistent with a model showing that cDC2s prime CD4 T cells. Additionally, in a tumor model, cDC2s induced CD4 T cell proliferation, and the generation of antitumor activity required depletion of regulatory CD4 T cells [135]. This result might imply that cDC2s prime CD4 T cells, but in this study, proliferation was examined after tumor implantation, and a role for proliferating CD4 T cells in a ‘helping’ capacity, such as for licensing cDC1s, was untested [135],. Nonetheless, the idea that cDC2s are the main APCs that prime CD4 T cell responses have been widely accepted as a general principle [135].

However, other evidence argues against a strict requirement for cDC2s in priming CD4 T cells in all settings. CD4 T cells are multifaceted cells and can acquire various effector programs; [136] therefore, equating proliferation with help may not be justified. Furthermore, there are counterexamples, as some studies have shown that cDC2s display a substantially lower capacity for processing cell-associated antigens than cDC1s [93, 137]. In fact, one study showed a much greater capacity for cDC1s than cDC2s for processing of MHC class II antigens, depending on the form of the antigen [93]. In the case of cell-associated antigens, cDC1s were found to be more efficient than cDC2s. This result suggested that cDC2s as the sole primers of CD4 T cells may not be universally true. In summary, it is not clear whether cDC1s or cDC2s are critical for priming CD4 T cells that are relevant to cDC licensing in the context of antitumor responses. A challenge to testing this possibility in vivo is presented by the lack of precise genetic models that can be used for selectively interrupting gene function in cDC1 and cDC2s.

## What is the basis for CD4 T cell help?

Studies suggesting a role for helper cells in enhancing responses of cytolytic T cells extend as far back as the 1970s [138–141] A major mechanism considered in this early period was based on cytokines, such as IL-2, that CD4 T cells can secrete to enhance CD8 T cell responses [142]. Later studies focused on different pathways [143–145], suggesting that interactions between CD40 expressed by CD4 T cells and CD40L expressed by target cells were involved in the mechanism of action. The primary targets considered for this interaction were APCs, such as DCs. Direct CD4 T cell:CD8 T cell interaction was proposed as a mechanism in the setting of T helper cells with CD8 memory T cells [146], although this supposition has been disputed. A series of studies focused on the impact of CD4 T cell help on CD8 T cell memory [147–150]. The dependence of CD8 T cell memory on CD4 T cell help varied with specific conditions, such as the dose of bacterial pathogen or the presence of TLR stimulation. However, these studies did not examine the requirement for CD4 T cell help in the setting of antitumor responses.

The issue of the target cell of CD4 T helper cells was addressed in later studies. cDC1s have been suggested as targets of CD4 T cells in an in vitro analysis of CD4 T helper cell-dependent CD8 T cell responses [151] and by intravital imaging during viral infection [152, 153]. CD40 ligation with CD4 T cells is a potential mechanism that augments CD8 responses. However, mechanisms in addition to the CD40 licensing of APCs [144, 145] have included the production of cytokines [154], such as IL-2; prevention of TRAIL expression in CD8 T cells’ [155], and direct activation of CD40 signaling in CD8 T cells [146]. However, for all of these proposed mechanisms, a direct demonstration of a cDC1 requirement for mediating the help provided by antigen-specific CD4 T cells has not yet been established in vivo.

## cDC1-specific CD40 signaling is required for optimal CD8 T cell responses

Evidence supporting a direct interaction between CD4 T cells and antigen: MHC-II complexes on cDC1 was recently obtained using a cDC1-specific Cre strain system [92]. First, a new system of cDC1 lineage ablation was used to establish a requirement for cDC1 in tumor rejection. Two problems with the original *Batf3*-deficient mouse system were that (1) *Batf3* is inactivated in multiple types of cells, including cDC2s, and (2) the cDC1 lineage can develop in some settings, especially those that generate IL-12 or IFNγ, which induce the expression of *Batf* and *Batf2* to compensate for the loss of *Batf3* [8]. However, molecular analysis has led to the ability to eliminate the cDC1 lineage without inactivating the *Batf3* gene. In mice with the deletion of a 400-bp region of the +32-kb *Irf8* enhancer, called *Irf8* + 32^*–/–*^ mice [59], cDC1 development is fully ablated without disrupting *Batf3*, and cDC1 cells are never reestablished even under conditions that induce compensatory cDC1 development in the *Batf3*^−/−^ mice. Tumor studies with these *Irf8* + 32^*–/–*^ mice firmly established that cDC1 itself, not Batf3 acting in other cells, is required for the rejection of tumors.

Next, work with a novel Xcr1-Cre strain was used to test the specific interactions involving cDC1 that occur during tumor rejection [92]. First, the conditional deletion of all MHC-I molecules from cDC1, using Xcr1-Cre crossed with a floxed allele of β2 microglobulin, confirmed the expected peptide: MHC interactions with CD8 T cells in tumor rejection. In addition, deletion of the MHC-II molecule I-A^b^ on cDC1 impaired tumor rejection, consistent with a role for antigen-specific CD4 helper T cell recognition of cDC1s. Unexpectedly, this study also showed a reduction in the early priming of CD4 T cells. When MHC-II was deleted only from cDC1s, CD4 T cell responses were substantially diminished. When cDC1s were the only cells expressing MHC-II, CD4 T cell priming continued. In conclusion, cDC1s are capable of priming CD4 T cells that participate in the licensing of cDC1s for enhanced CD8 T cell responses to tumors.

**Fig. 4:**
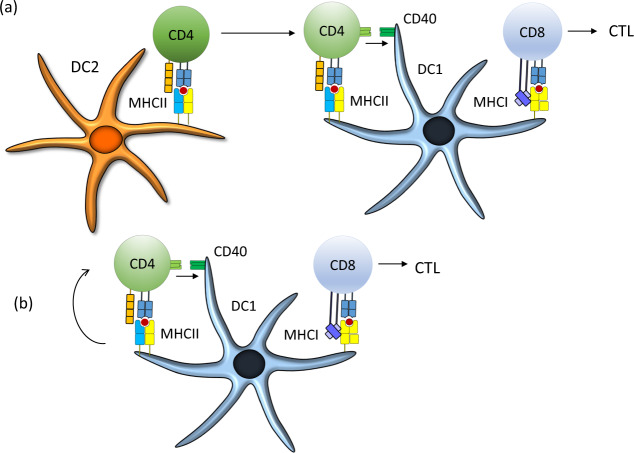
Revised model of CD4 T cell licensing of cDC1s to induce CD8 T cell responses. **a** In one model, CD4 T cells must be primed by cDC2s that have captured and presented tumor-derived antigens. These CD4 T cells migrate to the location of a cDC1 involved in priming CD8 T cells. **b** In a revised model, cDC1s serve as platforms for priming both CD4 T cells and CD8 T cells. The ligation of CD40 on the cDC1 surface during CD4 T cell priming is essential for strong licensing of cDC1s, and the lack of this ligation can lead to a failure in tumor rejection mediated by CD8 T cells. The mechanism downstream of CD40 signaling in cDC1s that leads to enhancement of CD8 responses is not currently known but may involve the induction of several costimulatory molecules, such as CD70 and 41BB, that augment the activation of CD8 T cells, as well as survival factors within the cDC1sthemselves.

These results did not exclude the possibility that cDC2s might be APCs for CD4 T cells, especially late in the response to tumors, as indicated by others [135]. However, a prevailing model suggests that CD4 T cells are first primed by cDC2s and then re-engage different APCs, such as cDC1, to license CD4 T cells and enhance the CD8 T cell response (Fig. 4a) [135, 156, 157]. This model was based on studies in which antigens were targeted to DCs using antibodies [131–133]. However, the results with the *Xcr1*-Cre model [92] suggested that cDC1s are sufficient platforms for priming CD4 T cells that then license them, particularly in the setting of tumors where the relevant antigens are cell-associated (Fig. 4b).

It is likely that the pathways involved in taking up and processing antigens differ for soluble proteins, cell-associated proteins captured by specific receptors (e.g., CLEC9A), and those targeted by antibodies. CLEC9A is a highly cDC1-specific receptor that binds filamentous actin and likely provides cDC1s with sufficient antigens from dead cells that they can efficiently expose on their intracellular membrane surfaces [158, 159]. In this process, antigens may be captured in distinct intracellular vesicles and delivered through other routes. These processes are active areas of research.

Naïve CD4 T cells constitutively express intracellular CD40L [160]. For this reason, even naïve CD4 T cells are poised to license cDC1s upon initial activation and thus can function as one arm of a ‘coincidence detector’ in responding to antigens presented by either MHC-II or MHC-I molecules. This two-part detection requirement adds a layer of security for fully activating CD8 T cell responses. It is possible that noncognate cDC licensing could occur, and some evidence for this was provided in an analysis of mice expressing the transpeptidase sortase A fused to CD40L [161]. In this study, after 12 h of immunization using peptide-loaded DCs, the enzymatic labeling of CD40 on DCs was found to require the expression of MHC-II. However, after 48 h, CD40 labeling occurred, to an extent, on cDCs that did not express MHC-II [161]. This finding indicates noncognate CD40 marking by the sortase and may represent noncognate DC licensing. However, whether the marked DCs were functionally licensed by the CD40 signal was not tested, and the possibility remains to be determine. When *Xcr1*-Cre was used to delete either MHC-II or CD40, tumor rejection was severely reduced, which suggests that a cognate interaction between CD4 T cells and cDC1s is required, at least to realize the full effect of CD8 T cell priming.

## Final comments

The developmental biology of conventional DCs relates to tumor immunology in a fundamental way, illustrating that the antitumor responses relying on molecular pathways in DCs are also directed toward defense against intracellular pathogens, especially viruses. Defenses against both tumors and viruses rely heavily on a cDC1 subclass, the *Batf3*-dependent type, a specialized cross-presenting cell, and on physiologic pathways integrating CD4 T cell activity to ‘license’ cDC1s for optimal CD8 T cell priming. While it is probably clear that cognate CD4 T cell interactions, via antigen recognition on cDC1s, and subsequent CD40 signaling in cDC1s are key parts of the pathway that enhance CD8 responses, the elements downstream of CD40 signaling in cDC1s that mediate this effect on CD8 T cells remain unknown. Ongoing work will likely reveal unexpected findings related to whether induction of specific costimulatory molecules, such as CD70 or 41BB, on cDC1s are sufficient. Further, the critical role of the BDCP WDFY4 in tumor rejection illustrates the variability of cells in processing antigens and the importance of using model systems that reflect the in vivo situation. It is fair to say that we truly know nothing about how WDFY4 executes cross-presentation except that it is required in vivo and in vitro by the cells that perform cross-presentation in vivo against tumors. To say anything more would be purely speculative. Regarding the mechanisms of CD4 T cell help and cDC1 licensing, we need to mention active research into the use of anti-CD40 antibodies to enhance antitumor responses [162]. The underlying mechanisms of this therapy are not completely known but may include direct actions on cDC1s. The analysis of cDC1 licensing in experimental systems and identification of the relevant targets of CD40 signaling in cDC1s may help in better understanding the basis for anti-CD40 antibody enhancement of ICT.
